# From promise to practice: CAR T and Treg cell therapies in autoimmunity and other immune-mediated diseases

**DOI:** 10.3389/fimmu.2024.1509956

**Published:** 2024-12-04

**Authors:** Yannick Bulliard, Robert Freeborn, Molly Javier Uyeda, Daryl Humes, Ryan Bjordahl, David de Vries, Maria Grazia Roncarolo

**Affiliations:** ^1^ Department of Research and Development, Tr1X, Inc., San Diego, CA, United States; ^2^ Division of Hematology, Oncology, Stem Cell Transplantation, and Regenerative Medicine, Department of Pediatrics, Stanford University School of Medicine, Stanford, CA, United States

**Keywords:** Tr1, Treg, autoimmunity, CAR T, cell therapy, Tr1 cells

## Abstract

Autoimmune diseases, characterized by the immune system’s attack on the body’s own tissues, affect millions of people worldwide. Current treatments, which primarily rely on broad immunosuppression and symptom management, are often associated with significant adverse effects and necessitate lifelong therapy. This review explores the next generation of therapies for immune-mediated diseases, including chimeric antigen receptor (CAR) T cell and regulatory T cell (Treg)-based approaches, which offer the prospect of targeted, durable disease remission. Notably, we highlight the emergence of CD19-targeted CAR T cell therapies, and their ability to drive sustained remission in B cell-mediated autoimmune diseases, suggesting a possible paradigm shift. Further, we discuss the therapeutic potential of Type 1 and FOXP3^+^ Treg and CAR-Treg cells, which aim to achieve localized immune modulation by targeting their activity to specific tissues or cell types, thereby minimizing the risk of generalized immunosuppression. By examining the latest advances in this rapidly evolving field, we underscore the potential of these innovative cell therapies to address the unmet need for long-term remission and potential tolerance induction in individuals with autoimmune and immune-mediated diseases.

## 1 Introduction to autoimmune and other immune-mediated diseases

### 1.1 Overview of autoimmune diseases and current treatment approaches

Autoimmune and inflammatory diseases, which encompass a wide range of disorders with complex pathology and etiology, affect between 5-10% of the population in industrialized regions, and their burden continues to increase (1). Although heterogeneous, autoimmunity generally arises from a combination of genetic predisposition and environmental co-factors leading to initiation and potentiation of attacks by the body’s own immune cells, specifically autoreactive T and B cells (2). Conventional first-line approaches for the treatment of autoimmune diseases have historically involved either the suppression of general immune function to modulate uncontrolled inflammation via corticosteroids or immunosuppressants such as azathioprine and methotrexate, or the use of disease-modifying anti-rheumatic drugs (DMARDs). This broad, non-specific efficacy, combined with lifelong treatment requirements, leads to frequently intolerable side effects, including increased risk of infection due to continued immunosuppression (3). More recently, biologics focused specifically on certain components of the immune system, such as cytokines or cell surface proteins, have been applied successfully to these types of diseases. Similar to steroids and DMARDs, these newer biologics and small molecules, which include TNF-alpha inhibitors, other monoclonal antibodies, and JAK or TYK2 inhibitors, target downstream inflammatory mediators but generally do not address the underlying disease drivers. Unfortunately, many patients eventually experience primary treatment failure or lose response to these therapies over time due to the development of anti-drug antibodies (4).

Autoimmune diseases are largely characterized by an aberrant immune response directed against self-antigens, often mediated by dysregulated T and B lymphocytes. T cells can inflict direct tissue damage or orchestrate inflammatory cascades via cytokine release (5), while B cells contribute to pathology through both production of pro-inflammatory cytokines and through production of autoantibodies, which can bind to self-antigens on tissues or form immune complexes leading to complement activation and inflammation (6). Therapeutic strategies aimed at depleting these pathogenic lymphocytes seek to interrupt the self-enhancing damage they cause and have demonstrated clinical success across several indications.

### 1.2 Dual B and T cell targeting: mechanistic rationale to treat autoimmunity

While not curative, lymphocyte depletion or suppression has demonstrated efficacy in managing a range of autoimmune conditions, albeit with a concomitant risk of opportunistic infections due to transient generalized immunosuppression. Rituximab, a chimeric monoclonal antibody targeting the CD20 antigen on B cells, exemplifies the clinical utility of B cell depletion in autoimmune disease management. It has been shown to reduce disease activity in rheumatoid arthritis, systemic sclerosis and multiple sclerosis, among other diseases (7). Conversely, abatacept, an IgG1/CTLA4 fusion protein, works to inhibit T-cell activation by binding to CD80 and CD86 on antigen-presenting cells, preventing their interaction with CD28 on effector T cells and is approved in several autoimmune indications including rheumatoid arthritis and psoriatic arthritis. Combination therapies, aiming to target both cell subsets, including using rituximab and abatacept simultaneously have been attempted in autoimmune diseases such as rheumatoid arthritis. The results have demonstrated good efficacy but a mixed safety profile due to broad immunosuppression and associated increased infection rate (8). This dual targeting approach is also being explored in trials evaluating anti CD20 therapies in combination with different cell therapies, including NK cells, as further described below.

Newer agents have sought to improve on the specificity and safety profile of previous generation biologics by focusing on the mechanisms and signaling driving inflammation, tissue damage and autoantibody production, rather than broadly depleting B cells or suppressing T cell proliferation. Specifically, antibodies targeting more mature B cells than anti-CD20 (9) or both mature B and T cell subsets (e.g. anti-CD52 antibodies) (10) are being investigated in a number of autoimmune disease settings. Further studies are ongoing to identify bi-specific antibodies capable of an even more robust and precise B cell depletion than traditional monoclonal antibodies, by driving improved lymph node and tissue penetration (11). Beyond depletion, therapeutic antibodies and small molecules that can act on B cell and T cell signaling have also gained traction in treating a wide range of autoimmune disorders, such as those focusing on intracellular pathways (e.g complement inhibitors, S1PR modulators, TYK2 inhibitors, IL-12/23p40 inhibitors, and JAK inhibitors, among others) (12–14). In particular, JAK inhibitors have been successful in tackling a number of autoimmune and inflammatory diseases across rheumatological, dermatological, gastrointestinal and transplant-related disorders. Their efficacy in these multiple settings is somewhat hindered by their less-than-ideal toxicity profile, including the increased risk of infections, cardiovascular risk, hematological effects and malignancies, resulting in a boxed warning from the FDA.

Crucially, not all patients respond equally well, both in terms of depth and duration of response within the same indication to the same class of drug. This highlights the importance of understanding both the unique pathophysiology of disease in each individual, and the need for identification of biological markers predictive of response in heterogeneous patient populations. A recent example is the use of a companion diagnostic to identify likely responders to anti-tumor necrosis factor-like cytokine 1A (TL1A) antibodies for patients with inflammatory bowel disease (15)

Yet, despite these improvements in precision and reduction in overall toxicity profile, these novel therapies still only serve to manage, rather than cure autoimmune and immune-mediated diseases. Cell therapies, in particular CAR-T cells, which involve the manipulation and administration of engineered T cells to treat disease, have revolutionized the treatment landscape in oncology and are now being adapted for autoimmune and inflammatory disorders. In addition to conventional CD3^+^ T cells, other cell therapies, including hematopoietic stem cell transplant (HSCT), regulatory T (Treg) cells (16), natural killer (NK) cells and mesenchymal stem cells (MSCs) are being investigated for their ability to modulate immune responses and promote tolerance. These therapies aim to restore immune homeostasis by either suppressing autoreactive immune cells or promoting regulatory pathways that prevent tissue damage (17). The goal of these therapies is to induce long-term remission via the restoration of immune tolerance.

## 2 Cell therapies in autoimmunity and immune-mediated diseases: a new frontier

### 2.1 HSCT: the last line of defense

The use of cell therapy to treat autoimmune diseases traces back to the 1990s with the first confirmed use of hematopoietic stem cell transplantation (HSCT) for connective tissue disease and severe pulmonary hypertension (18). Since those early cases, HSCT has evolved to become a viable option for various severe, treatment-resistant autoimmune conditions, including multiple sclerosis, systemic sclerosis, and Crohn’s disease (19). HSCT involves the replacement of a patient’s hematopoietic and immune system with stem cells from a healthy donor (allogeneic HSCT), or more commonly in autoimmune diseases, from the patient themselves (autologous HSCT).

The mobilization and collection of peripheral stem cells for HSCT is a multi-step process that requires careful monitoring and management. Mobilization of stem cells from the bone marrow of patients into the peripheral blood is achieved primarily through the administration of granulocyte colony-stimulating factor (G-CSF), or in combination with cyclophosphamide as pre-treatment (20). Once mobilized, the stem cells are collected from the peripheral blood via leukapheresis. After collection, these cells can undergo further processing or manipulation based on the specific HSCT protocol being followed. Various protocols are currently used, and the optimal protocol for maximum therapeutic benefit has not been definitively established. For instance, in patients with systemic sclerosis treated with autologous HSCT, enrichment of CD34+ progenitor cells does not appear to provide additional clinical benefit (21).

The rationale behind HSCT in autoimmune diseases lies on the potential to ‘reset’ the aberrant immune system, fostering the regeneration of a self-tolerant immune repertoire (22). The precise mechanisms by which HSCT orchestrates immunological reset remain an area of active investigation. The process involves a multi-faceted interplay of immune cell depletion via lymphoablative conditioning, lymphopenia-induced proliferation, differentiation of new T cell progenitors in the thymus, and the modulation of Treg cells (23). Importantly, Treg cells, key players in immune tolerance, undergo significant changes following HSCT, with studies reporting an increase and more diverse Treg cell repertoire after autologous HSCT. This is associated with greater suppressive activity in immune mediated diseases such as Crohn’s, multiple sclerosis, systemic lupus erythematosus (SLE), and systemic sclerosis (24–28). In the context of allogeneic HSCT, a high frequency of donor Treg cells has been associated with a lower risk of Graft-versus-Host Disease (GvHD) mediated by donor effector T cells, which remains a problematic and frequently lethal side effect of HSCT (29).

Importantly, the procedure is not without risks. HSCT is an onerous process for patients that involves multiple complexities, including the need for taxing immunoablative pre-conditioning regimens, which increases treatment-related morbidity and mortality due to severe infections after immunosuppression and cytopenia (30). In the last decade, the safety of the procedure has shown remarkable improvement, thanks to better selection of the most appropriate patients to transplant, better donor matching, and decreased intensity of conditioning regimens, including use of novel agents. For patients with a relapse-remitting form of multiple sclerosis for instance, treatment related mortality due to autologous HSCT has decreased to <1% (31). For systemic sclerosis, where treatment-related mortality mostly stemmed from cardiac complications, careful patient selection has enabled a decrease mortality rate to <6% (19). While less frequently used in autoimmune disease compared to oncology, HSCT can be with is only really considered as a treatment option for life-threatening forms of these diseases or relapsing courses of disease occurring after autologous transplantation. Allogeneic HSCT carries a greater risk of death and complications compared to autologous HSCT (23). Thus, though HSCT has demonstrated benefit in treating conditions including multiple and systemic sclerosis, its use remains restricted to a limited number of patients with severe disease who have not responded to conventional or approved therapies.

### 2.2 Mesenchymal stem cell therapies

Research into the immuno-modulating and regenerative properties of MSCs has sparked interest in this primarily allogeneic stem cell-based modality for the treatment of autoimmune diseases (32). MSCs are believed to exert their therapeutic effects through paracrine signaling, releasing cytokines that can suppress pro-inflammatory responses, promote tissue repair, and modulate immune cell function (33). MSCs can also be engineered to deliver a therapeutic payload. For instance, IL-10-expressing MSCs have been shown to decrease inflammation and improve fine motor function after traumatic brain injury in rats (34). Clinical trials investigating the use of MSCs in autoimmune diseases such as Crohn’s disease and ulcerative colitis (35–37), GvHD (38), multiple sclerosis (39, 40) and severe idiopathic nephrotic syndrome (41) have so far reported encouraging, albeit mixed results. Likely contributors to these mixed results include the heterogeneity of MSC populations across tissues (42), the variability in isolation and expansion protocols (43), their limited persistence and trafficking (44), and uncertainty regarding the optimal route and dosing regimens to use across indications (45).

### 2.3 Moving beyond stem cells

The utilization of stem cells of hematopoietic or mesenchymal origin in autoimmune diseases, while groundbreaking, has encountered limitations such as high toxicity and inconsistent efficacy in a limited number of patients. Moreover, these therapies are not specifically tailored to target or eliminate disease-inducing immune cell subsets, such as autoreactive B or T lymphocytes. These limitations have spurred the exploration of alternative cell-based therapies that offer a more targeted and potentially safer approach to modulating the immune system in Treg cells and conventional T cells armored with a B-cell targeting moiety, such as a CD19-targeting chimeric antigen receptor (CAR).

While outside the scope of this review, additional differentiated cell subsets are also in development, such as NK cell therapy, an emerging modality in cancer immunotherapy and autoimmune disease. NK cells are innate immune effector cells that possess potent cytolytic function while exhibiting minimal risk of GvHD in the context of an allogeneic, donor-derived therapeutic due to both low allo-reactivity and limited persistence. Thus, NK cells do not have the need for strict HLA matching, a limitation of allogeneic effector T cell-based therapies. Engineering NK cells to express CARs targeting autoantigens or B-cell specific receptors enable precise targeting and elimination of pathogenic cell populations (46, 47). The efficacy of NK-based cell therapy, both with and without CAR modifications, in treating autoimmune diseases like SLE is currently being investigated in ongoing clinical trials both as a standalone therapy and in combination with more traditional B cell depleting agents. The results of these trials will provide valuable insights into the potential of this therapeutic approach for managing immune-mediated conditions.

## 3 CAR T cells to treat autoimmunity

### 3.1 CAR and TCR T cell therapy in oncology

The development of CAR T cells engineered to target the CD19 cell-surface antigen has revolutionized the treatment of B cell malignancies. Clinical trials have demonstrated their remarkable efficacy, with complete response rates ranging from 40-54% in multiple B cell lymphoma subtypes (48–50), 71-81% in B cell acute lymphoblastic leukemia (51, 52) and 18% in chronic lymphocytic leukemia (53). The durability of these responses suggests curative potential for some patients receiving these cells. The success of CD19-targeted CAR T cells has led to their approval by the FDA, either as a standalone therapy or as a bridge to HSCT (Axicabtagene ciloleucel, Tisagenlecleucel, Lisocabtagene maraleucel, Brexucabtagene autoleucel). In addition, CAR T cells targeting BCMA (Idecabtagene vicleucel, Ciltacabtagene autoleucel) have demonstrated efficacy in difficult to treat multiple myeloma, further expanding the therapeutic landscape for CAR T cell therapy among hematological cancers. Development of TCR-engineered T cells and tumor-infiltrating lymphocyte (TIL)-derived therapeutics have followed closely behind CAR T cell development. Recently, a MAGE-A4-targeted TCR-T cell therapy was granted accelerated approval by the FDA for the treatment of synovial sarcoma (uzatresgene autoleucel; NCT03132922) (54). Likewise, in 2024, approval was granted to a patient-derived TIL cell therapy for the treatment of melanoma (lifileucel; NCT02360579) (55).

### 3.2 CAR T cell therapy in autoimmunity

Application of CAR T cells in autoimmunity has largely followed the path laid out by hematological malignancies, namely using autologous CAR T cells targeting BCMA and CD19 antigens on the surface of B cells. The rationale for employing B-cell targeted CAR T cell therapy in autoimmune diseases is grounded in the critical role played by pathogenic B cells and autoreactive antibodies in the pathogenesis of many such conditions. The aim of B-cell targeted CAR T cell therapy is to disrupt these pathogenic processes and restore immune balance by depleting both circulating B cells, as well as those in the tissues and lymph nodes that are not reached by traditional anti-CD20 therapies. Early clinical trials have yielded promising results, demonstrating the safety and efficacy of B-cell targeted CAR T cell therapy in various autoimmune diseases, including SLE, myasthenia gravis, idiopathic inflammatory myopathy, systemic sclerosis, neuromyelitis optica spectrum disorder, and multiple sclerosis (56) (**Tables 1**, **2**) (56). In a study involving five patients with refractory SLE who received anti-CD19 CAR T cell therapy, all patients achieved durable drug-free remission and cessation of nephritis (61). Mild cytokine release syndrome (CRS) occurred in all patients, with no concomitant immune effector cell-associated neurotoxicity syndrome (ICANS). Importantly, the B cell compartment reconstituted in these patients over a median of 8 months without disease flare or relapse. A follow-up study evaluating this product in fifteen severely ill patients with various autoimmune diseases, including eight with SLE, four with systemic sclerosis and three with inflammatory myopathies further confirmed these preliminary results. At a median follow-up of fifteen months, all the SLE patients were in drug-free remission without symptoms (62). The three treated patients with myositis also showed a major clinical response at six months, while the four patients with systemic sclerosis experienced decreases in disease severity scales and successfully discontinued all immunosuppressive medications during the follow-up period. Most autoantibodies were undetectable during up to twenty nine months of follow-up. Separately, in a Phase 1b/2a clinical trial of an anti-BCMA mRNA CAR T therapy in patients with generalized myasthenia gravis, eleven patients received six outpatient cell infusions (70). Patients receiving weekly infusions showed clinically meaningful benefits across three established clinical scoring systems and responses were maintained through the six-month follow-up. Serum titers of anti-AChR autoantibodies decreased by 22% at week 8 in patients with anti-AChR+ disease. Confirming these initial promising datasets, another anti-BCMA CAR T therapy was studied in twelve patients with neuromyelitis optica spectrum disorder (68). Eleven of twelve patients exhibited drug-free remission with no relapse during a median 5.5 months of follow-up. It is important to note that in these trials, all patients received lymphodepletion chemotherapy before CAR T infusion, and all experienced Grade 1 or 2 CRS.

**Table 1:** Ongoing clinical trials using CAR T Cell therapies in autoimmune diseases.

Sponsor	Product Name	Product Class	Product Type	Indication	Phase	Clinical Trial Number
**Adicet Bio​**	ADI-001​	Allogeneic	CD20 γδ CAR T​	LN​ & SLE	Phase 1​	NCT06375993
**Atara Bio​**	ATA3219	Allogeneic	EBV+ CD19-1XX CAR T​	LN​, SLE	Phase 1​	NCT06429800
**Autolus​ Tx**	Obe-cel​	Autologous	CD19 CAR T​	SLE​	Phase 1​	NCT06333483
**Bioray Lab​**	BRL-301​ TyU19​ ​	Allogeneic	CD19 CART + HLA-A/B, HLA-II, TRAC, PD1 KO	SLE​	Phase 1​	NCT05988216
				SLE; SSc; IM; Sjogren’s Syndrome​	Phase 1	NCT05859997
**BMS ​**	CC-97540​	Autologous	CD19 NEX T​	Multiple Sclerosis​	Phase 1​	NCT05869955
		Autologous	CD19 NEX T​	SLE​, IIM, SSc	Phase 1​	NCT05869955
**Cabaletta Bio​**	CABA-201​	Autologous	CD19 CAR T​	MG	Phase 1​	NCT06359041
				Myositis​	Phase 1​	NCT06154252
				SLE​	Phase 1​	NCT06121297
				Systemic Sclerosis​	Phase 1​	NCT06328777
				Pemphigus Vulgaris​	Phase 1​	NCT04422912
	DSG3-CAART	Autologous	Chimeric Auto-Antibody Receptor T cells​	Pemphigus Vulgaris​	Phase 1​	NCT04422912
	MuSK-CAART​	Autologous	Chimeric Auto-Antibody Receptor T cells​	MG	Phase 1​	NCT05451212
**Caribou​ Bio**	CB-010​	Allogeneic	CD19 CAR T​	LN​, ERL	IND cleared	n.a.
**Cartesian Tx​**	Descartes-08​	Autologous	BCMA mRNA CAR T​	Myasthenia Gravis​	Phase 2​	NCT04146051
				SLE​	Phase 2​	NCT06038474
**Essen Biotech​**	N/A	Autologous	CD19 BCMA CAR T​	Sjogren’s Syndrome​, SLE​	Phase 1/2	NCT06350110
**Fate Tx​**	FT819​	Allogeneic	CD19 CAR T​	SLE​	Phase 1​	NCT06308978
**Friedrich-Alexander University**	N/A	Autologous	CD19 CAR T​	SLE​	Phase 2​	n.a.
				SSc​	Phase 1​	n.a.
				IIM (Antisynthetase syndrome)​	Phase 1​	n.a.
**Gracell Bio​/AstraZeneca**	GC012F​	Autologous	CD19/BCMA CAR T​	SLE​	Phase 1​	NCT05846347
**IASO/Innovent​** **​**	CT103A​	Autologous	BCMA CAR T​	NMOSD, MG, IMNM	Phase 1​	n.a.
				NMOSD, MG, CIDP, IIM, MS, AE, MOGAD, POEMS Syndrome	Phase 1​	NCT04561557
**iCell Gene Tx​**	BCMA-CD19 CAR T​	Autologous	CD19/BCMA CAR T​	SLE​	Phase 1​	NCT05474885
**Immpact Bio​**	IMPT-514​	Autologous	CD19/CD20 CAR T	SLE, LN​	Phase 1/2	NCT06153095
				SLE, AAV, IIM​	Phase 1​	NCT06462144
				MS​	IND cleared	n.a.
**JW Tx​/BMS**	Relma-cel​	Autologous	CD19 CAR T​	SLE​	Phase 1​	NCT05765006
**Kyverna Tx**	KYV-101​	Autologous	CD19 CAR T	MS​	Phase 2​	NCT06384976
				LN​	Phase 1/2	NCT06152172
				IIM, SSc, SLE, AAV	Phase 1​	NCT06152172
				MG	Phase 2​	NCT06193889
				Myositis and myopathies​	Phase 1​	NCT06152172
				Stiff Person Syndrome ​	Phase 2	NCT06588491
				SSC	Phase 1/2	NCT06152172
**Luminary Tx​**	LMY-920​	Autologous	BAFF CAR T​	SLE​	Phase 1​	NCT06340750
**Miltenyi Biomedicine​**	MB-CART19.1​	Autologous	CD19 CAR T​	SLE​	Phase 1​	NCT06189157
**Novartis​**	YTB323​ (Rap-cel)	Autologous	CD19 CAR T​	SLE​	Phase 1/2	NCT05798117
**Renocell​**	RY_SW01​	Allogeneic	MSC​	LN	Phase 2​	NCT06058078
				SSC	Phase 1/2	NCT06489652
**Sana Bio**	SC291​	Allogeneic	CD19 CAR T	LN, SLE, AAV	Phase 1​	NCT06294236
**Synthekine**	SYNCAR-001	Autologous	CD19 CAR T + orthogonal IL2	SLE, LN​	Phase 1​	NCT06544330

SLE, systemic lupus erythematosus; LN, lupus nephritis; SSc, systemic sclerosis; IM, inflammatory myopathy; IIM, idiopathic inflammatory myopathy; ERL, extrarenal lupus; NMOSD, Neuromyelitis optica spectrum disorder; MG, myasthenia gravis; IMNM, immune-mediated necrotizing myopathy; CIDP, chronic inflammatory demyelinating polyneuropathy; AE, autoimmune encephalitis; MOGAD, myelin oligodendrocyte glycoprotein antibody disease; AAV, ANCA-associated vasculitis.

**Table 2:** Published clinical results for CAR T cell therapies in autoimmunity.

Institution	Target Antigen	Indication(s)	Pts #	Key Clinical Results	Reference
**Ruhr-University Bochum**	CD19	Myasthenia Gravis, Lambert Eaton Myasthenic Syndrome	2	Full mobility restored at 4- and 6-months post-CAR T	(57)
**Otto-von-Guericke University**	CD19	Myasthenia Gravis	1	Restoration of mobility at 62 days post-CAR T	(58)
**Friedrich-Alexander University**	CD19	Antisynthetase syndrome	1	Drug-free remission at 150 days follow up post-infusion	(59)
**University Medical Center Hamburg-Eppendorf**	CD19	Multiple Sclerosis	2	EDSS score decreased from 4.5 to 4 at 100 days follow up (n=1) or remained stable at day 28 follow up (n=1)	(60)
**Friedrich-Alexander University**	CD19	SLE	5	Drug-free remission maintained to median 12 month follow up in all patients	(61)
	CD19	SLE	8 (follow-up study)	100% of patients reached complete remission, with disease activity absent up to 29 months post-CAR T	(62)
	CD19	Idiopathic inflammatory myositis	3	Normalized muscular function	(62)
	CD19	Systemic Sclerosis	4	EUSTAR activity index decreased at 6 months post CAR T	(62)
	CD19	Systemic Sclerosis	1	Reduced disease activity indicated by EUSTAR activity index maintained through 6 month follow up	(63)
**University Hospital Tübingen**	CD19	Anti-synthetase Syndrome	1	Significant decrease in physician’s global assessment of disease activity at 150 days follow up.	(64)
**Shanghai Jiao Tong University**	CD19	Sjogren’s Syndrome with concurrent diffuse large B-cell lymphoma	1	Normal levels of ANA, anti-Ro-52, and cytokines and the improvement of dry mouth symptoms, without the use of glucocorticoids or tocilizumab	(65)
**Shanghai Changzheng Hospital**	CD19 (allogeneic)	Myositis and systemic sclerosis	3	Complete remission at 6 months follow-up in 3/3 pts	(66)
**Huazhong University of Science and Technology**	BCMA	Anti-SRP necrotizing myopathy	1	Disease improvement maintained at 18 months	(67)
	BCMA	Neuromyelitis optica spectrum disorder	12	92% drug-free remission with 11/12 patients relapse-free at median follow-up of 5.5 months	(68)
**Zhongshan City People’s Hospital**	BCMA-CD19	SLE + lupus nephritis	13	Symptom and medication-free remission maintained at 46 months for 10/10 evaluable patients	(69)

EDSS, expanded disability status scale; EUSTAR, European scleroderma trials and research group. Pts #, number of patients reported.

Of note, CD19-targeted CAR T cells appear to be more effective compared to other systemically administered B cell depleting agents like rituximab and obinutuzumab, in SLE and lupus nephritis (9, 71, 72). This difference in efficacy could be attributed to several factors: CD20, the target of rituximab and obinutuzumab, has a more restricted expression pattern along the B cell development axis compared to CD19 (73). Thus, the broader expression of CD19 allows CD19-targeted CAR T cells to deplete a wider range of B-cell populations, including CD19+CD20- plasma cells. These cells may play an important role in the pathogenesis of certain diseases, as they have been implicated in increased disease severity in preclinical models of multiple sclerosis (74). Additionally, the ability of CAR T cells to expand upon antigen-encounter, infiltrate tissues and induce deep *in situ* B-cell depletion, including tissue-resident B cells that are less sensitive to anti-CD20 antibody-mediated depletion, may contribute to their enhanced efficacy (75–77). The use of a lymphodepleting conditioning regimen prior to CAR T infusion may also contribute to deeper B cell depletion than antibodies alone.

Although CD19-targeted CAR T cell therapy effectively eliminates most autoantibodies against DNA and nucleosomal structures in SLE, certain autoantibodies, like those against Ro60 and Scl-70, persist (62). A possible hypothesis in need of confirmation is that B cell subsets responsible for their production do not express CD19. Despite a broader range of CD19 expression across B cell lineages compared to CD20, CD19-targeted CAR T cell therapies preserve the protective effect of prior vaccination in patients (62), possibly due to the preservation of long-lived, CD19-negative plasma cells responsible for vaccine-induced humoral immunity (78).

BCMA, expressed on mature B cells and plasma cells, presents another promising target for conditions where autoantibody production is driven by CD19-negative, BCMA^+^ plasma cells. Depleting BCMA-expressing cells with CAR T cell therapy has shown encouraging efficacy in neuromyelitis optica spectrum disorder, anti-signal recognition particle (SRP) necrotizing myopathy, and SLE (67, 68, 79). BCMA-targeted therapies may lead to a more extensive B-cell depletion and a greater alteration of the autoantibody repertoire, possibly increasing clinical benefit compared to CD19 CAR T (80). However, this broader targeting also results in a more significant depletion of protective antibodies, increasing the rate of infections and impairing pre-existing vaccine responses (81–83). BCMA CAR T cell therapy recipients have lower seroprotection to vaccine-preventable infections compared to CD19 CAR T-cell recipients, likely due to depletion of antibody-producing plasma cells (82). In a study investigating the impact of teclistamab, a BCMA-targeted T-cell engager antibody, on humoral immunity, the therapy was found to reduce the levels of polyclonal immunoglobulins and impair humoral immune response after vaccination (81). The high rate of serious infections in this patient group was attributed, at least in part, to the development of hypogammaglobulinemia and failure to generate new humoral immune responses. The use of IVIG supplementation was associated with a significantly lower risk of serious infections, supporting the use of immunoglobulin supplementation as primary prophylaxis in patients receiving a BCMA-targeting therapies. The development of bi-specific CAR T cells capable of targeting both CD19 and BCMA aims to further enhance B-cell depletion and prevent antigen escape (69, 84), but likewise might further elevate the risk of severe infections due to profound B cell depletion and suppressed vaccine-induced immunity.

To improve specificity, chimeric autoantigen receptor (CAAR) T cells have also been developed, intending to directly target autoantibody-producing pathogenic B cells. This is achieved by replacing the CAR’s targeting moiety with the autoantigen itself, while keeping the architecture of the CAR intracellular domain intact, in theory leading to the specific elimination of those B cells (85). CAAR T cell therapy is particularly relevant for diseases driven by well-defined autoantigens, such as muscle-specific tyrosine kinase (MuSK) in myasthenia gravis or desmoglein 3 (DSG3) in pemphigus vulgaris (86, 87). This approach is currently being tested in two clinical trials (NCT04422912, NCT05451212). It is important to note, however, that CAAR T cells primarily target B cells expressing a membrane-bound B-cell receptor (BCR) and may not be effective against diseases driven by long-lived plasma cells devoid of a BCR (86).

(85) In contrast to autoimmune diseases with a dominant pathogenic B cell component, chronic inflammatory diseases such as psoriasis and other inflammatory skin diseases, inflammatory bowel disease, and spondyloarthritis are unlikely to respond to B cell-targeted CAR T cell therapies. Indeed, these diseases are instead characterized by dysregulated T cell activation and IL-23/IL-17-mediated inflammation (88–90). Therefore, T cell-directed CAR T cells, such as those targeting the T cell-specific antigens CD5 and CD7, represent a promising alternative for patients with these predominantly T-cell mediated diseases. Several such products are currently undergoing clinical evaluation for the treatment of T cell malignancies with encouraging results (91–94), while their efficacy in autoimmune and chronic inflammatory diseases remains to be demonstrated.

A key limitation of current CAR and CAAR T cell therapies is their inability to simultaneously target multiple pathogenic cell types. This constraint poses a challenge in treating autoimmune diseases where the underlying pathophysiology involves a complex interplay of dysregulated B and T lymphocytes, along with inflammation driven by myeloid-derived innate immune cells. For example, while ANCA-associated vasculitis and myasthenia gravis both have a clear B cell component, neutrophils and thymus-derived T cells also play critical roles in their pathogenesis (95, 96). Similarly, in multiple sclerosis, while autoreactive B cells contribute to disease pathology, as evidenced by the efficacy of B cell-depleting antibodies (97, 98), definitive disease-causing autoantibodies have not been identified. Instead, a complex interplay between pathogenic B cells, autoreactive T cells, and CNS-localized inflammation is believed to drive demyelination, neurotoxicity, and disease progression (99–101). As discussed in the next section, FOXP3^+^ and Tr1-based Treg cell therapies are well-suited to dampen T cell-driven autoimmunity and inflammation, but lack the ability to specifically deplete B cells. Combining these therapies with B cell-modulating agents, such as anti-CD20 antibodies or Bruton tyrosine kinase (BTK) inhibitors, may represent the next step in the evolution of these treatments, conferring multimodal activity for complex autoimmune diseases. Crucially, any combination therapy must maintain an acceptable safety and tolerability profile without inducing generalized immunosuppression that could increase the risk of opportunistic infections.

## 4 Regulatory T cells to treat immune-mediated diseases

**Figure 1:**
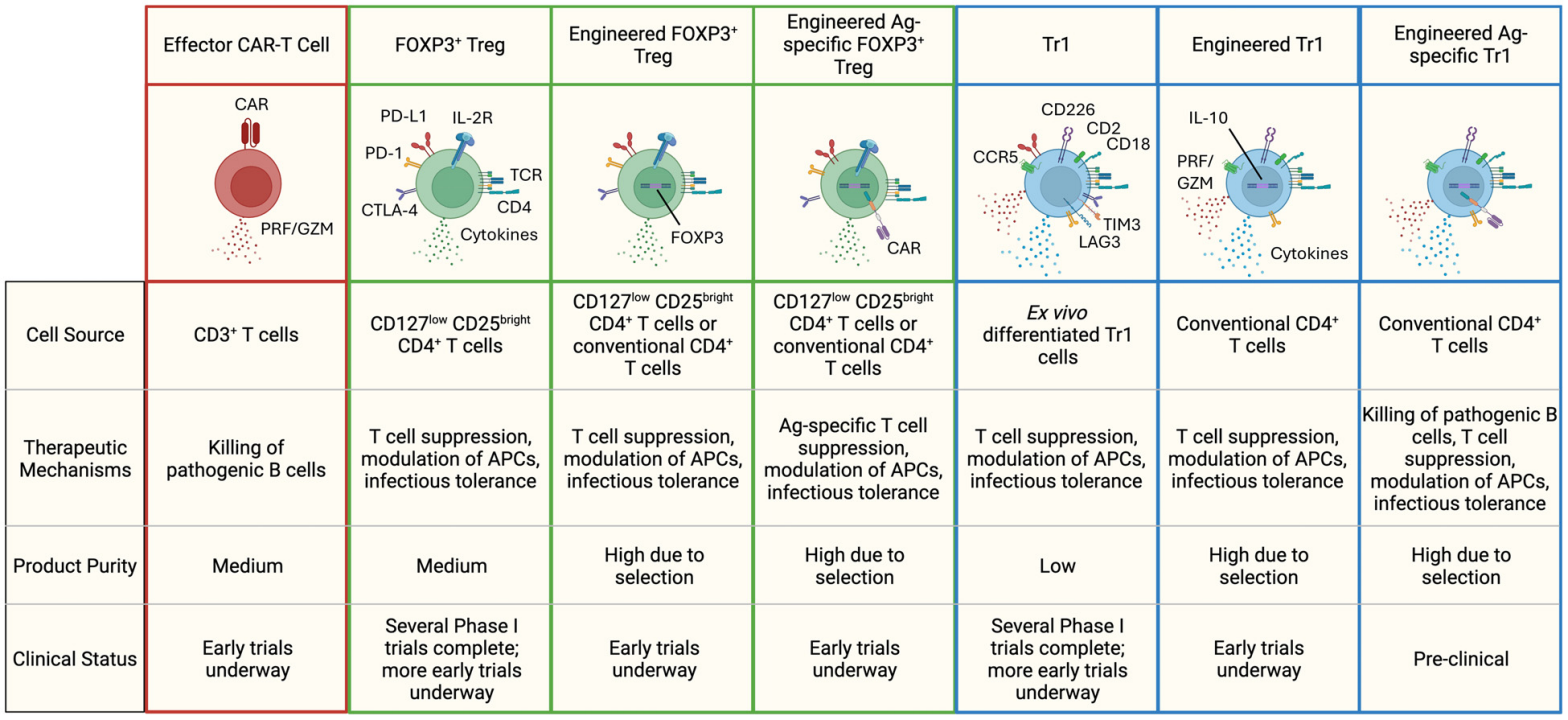
Different types of T-cell based cell therapies for the treatment of autoimmune diseases. Highlighted are some of the hallmark features of each cell therapy subset. FOXP3^+^ Tregs secrete immuno-regulatory cytokines like TGF-β and IL-35, while Tr1 cells produce high levels of IL-10 and TGF-β, along with moderate amounts of IFN-γ, IL-22, and IL-5. FOXP3^+^ Tregs are identified by elevated expression of the high-affinity IL-2 receptor and co-inhibitory receptors (PD-L1, PD-1, CTLA-4). Tr1 cells exhibit higher levels of CCR5, CD2, CD18, CD226, and co-inhibitory receptors (LAG-3, PD-1, TIM-3, CTLA-4, PD-L1). In therapies lacking an engineered surface marker (e.g., CAR, truncated-non-signaling forms of NGFR or EGFR), a combination of markers is used to define product purity. PRF, perforin; GZM, granzymes.

Regulatory T cells represent a diverse subset of lymphocytes responsible for maintaining immunological tolerance and homeostasis to both self and foreign peptides in healthy individuals. Treg cells are frequently underrepresented or dysregulated in patients with autoimmune conditions. Here we highlight the immunomodulatory mechanisms of two major CD4^+^ regulatory T cell types: FOXP3^+^ Treg cells and Tr1 Treg cells, and how their functionality can be harnessed to treat autoimmunity (**Figure 1**).

### 4.1 Immunosuppressive functionalities of FOXP3^+^ and Tr1 Treg cells

FOXP3^+^ Treg cells are primarily identified by their robust expression of the transcription factor Forkhead Box Protein 3 (FOXP3) alongside high levels of the high-affinity IL-2 receptor α (CD25) and low expression of the IL-7 receptor (CD127) (102). Additional markers associated with FOXP3^+^ Treg cells include elevated expression of the transcription factor Helios (103), high levels of the co-inhibitory surface molecule CTLA-4 (104), and specific demethylation patterns within the Treg-specific demethylation region (TSDR) (105). These phenotypic attributes underpin the potent immunomodulatory functions of FOXP3^+^ Treg cells, which are mediated through a combination of indirect and direct mechanisms (106) and include:

1. IL-2 deprivation: FOXP3^+^ Treg cells express high levels of CD25, enabling them to efficiently consume IL-2, thereby limiting the availability of this cytokine for effector T cells and curbing their proliferation (107).

2. Co-inhibitory signaling: FOXP3^+^ Treg cells express co-inhibitory receptors such as CTLA-4, which can modulate APCs and alter their antigen-presentation functionality (108).

3. Adenosine production: Through the sequential action of the ectoenzymes CD39 and CD73, FOXP3^+^ Treg cells convert extracellular ATP to adenosine, an immunosuppressive molecule that promotes a tolerogenic environment (109).

4. Inhibitory cytokine secretion: FOXP3^+^ Treg cells secrete a range of inhibitory cytokines, especially TGF-β and IL-35, which suppress immune cell activation and foster anti-inflammatory responses (110, 111).

The critical role of FOXP3^+^ Treg cells in immune regulation is underscored in disease states where their function or presence is compromised. For instance, patients harboring mutations in the FOXP3 gene are diagnosed with immune dysregulation, polyendocrinopathy, enteropathy, X-linked (IPEX) syndrome (112). These individuals often exhibit a constellation of autoimmune manifestations, including enteropathy, type 1 diabetes, and dermatitis (113). In other autoimmune diseases with more complex etiologies, such as SLE (114) and rheumatoid arthritis, the relationship between FOXP3^+^ Treg cells and disease pathogenesis is less clear. Studies have reported inconsistent findings regarding FOXP3^+^ Treg cell phenotype and frequency in these conditions, with observations ranging from increased to decreased numbers, to no discernible changes compared to healthy donors or in relation to disease severity (115), though there is clear evidence of FOXP3^+^ Treg dysregulation (116). Whether FOXP3^+^ Treg defects are a causative factor in these systemic autoimmune diseases, a consequence of the ongoing inflammatory process, or a combination of both, remains an area of active investigation (115, 117).

Tr1 Treg cells are a distinct subset of regulatory T cells that do not constitutively express high levels of the transcription factor FOXP3. These cells are induced in the periphery and play a crucial role in maintaining tolerance to non-pathogenic antigens. Tr1 Treg cells are characterized by the co-expression of the surface markers CD49b and LAG3, and the production of high levels of IL-10 and IL-22, with low levels of IL-2 and IL-4 (118). IL-10, a key immunoregulatory cytokine produced by Tr1 Treg cells, can suppress the activation and proliferation of effector T cells directly, or indirectly by downregulating the function of antigen-presenting cells (119–121). Furthermore, high IL-10 secretion by Tr1 Treg cells has been shown to downregulate the NLRP3 inflammasome in monocytes, reducing the production of pro-inflammatory cytokines such as IL-1β (122). IL-22 secretion by Tr1 cells contributes to the protection of epithelial barrier integrity from inflammatory damage (123). In addition to cytokine-mediated suppression, Tr1 Treg cells also exert cell-to-cell contact-mediated suppression through the engagement of co-inhibitory receptors such as PD-1, CTLA-4, and TIM-3 (124).

Tr1 cells were first identified in patients with severe combined immunodeficiency (SCID) receiving mismatched HSCT (125). These patients showed long term engraftment of donor-derived T cells with no GvHD, despite complete HLA-mismatch, providing evidence for peripherally-induced tolerance. Subsequent studies on patient-derived material identified antigen-specific IL-10-producing T cells, later termed Tr1 Treg cells, as crucial in achieving transplant tolerance (126, 127). Dysregulated Tr1 Treg cell function has been strongly associated with autoimmune and inflammatory diseases such as multiple sclerosis, Crohn’s Disease, and type 1 diabetes (128). Studies characterizing the immune systems of patients with these diseases consistently show a reduced frequency or impaired suppressive function of Tr1 Treg, or IL-10-producing CD4^+^ T cells, compared to healthy individuals (128).

### 4.2 Ex-vivo expanded FOXP3^+^ Treg cells as therapy to treat immune-mediated diseases

The immunomodulatory properties of regulatory T cells have generated considerable interest in their use as a therapeutic. However, harnessing their potential faces challenges, including the low frequency of FOXP3^+^ Tregs in the blood, which complicates their isolation and expansion for therapeutic use. Manufacturing polyclonal FOXP3^+^ Treg-based cell therapies typically involves a multi-step process: first, Treg cells are isolated from peripheral blood based on surrogate cell surface markers such as CD4, CD127, and CD25. This step may also include the depletion of CD8^+^ T or CD19^+^ B lymphocytes. Next, the isolated FOXP3^+^ Treg cells are activated and expanded *in vitro*, typically using anti-CD3 and anti-CD28 antibodies in the presence of high-dose IL-2 (129). Rapamycin, an mTOR inhibitor, which has been shown to promote the expansion of FOXP3^+^ Tregs, is often added to selectively promote Treg expansion while limiting the growth of contaminating non-Treg cells (130, 131).

Early clinical trials have employed *ex-vivo* expanded polyclonal FOXP3^+^ Treg cells for the prevention or treatment of GvHD in the context of allogeneic HSCT, or graft rejection in the context of solid organ transplants. In 2009, a first of its kind clinical study used ex-vivo expanded polyclonal FOXP3^+^ Treg cells for the treatment of GvHD (132). Another study of polyclonal FOXP3^+^ Treg cells in kidney transplant proved safe, with increased levels of circulating Treg cells following the cell therapy’s administration (NCT02145325) (133). In the context of solid organ transplantation, donor-antigen-specific FOXP3^+^ Treg cells, generated by activation with donor-derived antigen-presenting cells, have shown superior efficacy in promoting allograft survival in preclinical models compared to polyclonal FOXP3^+^ Treg cells (134–137). A clinical study involving liver transplant patients receiving donor-specific Treg cells demonstrated the safety of this approach, with some patients able to discontinue immunosuppressive drugs for over 24 months (138). An alternative method of activation, distinct from CD3 or APC stimulation, utilizes the HIV envelope glycoprotein gp120’s ability to stimulate these cells (139). The interaction of gp120 with CD4 on Treg cells triggers their activation and boosts their suppressive function, demonstrating protection against GvHD in humanized mice. This approach (Actileucel) has progressed to clinical trials, with the first patient treated earlier this year in a GvHD prevention trial. Beyond GvHD, multiple trials have assessed or are actively investigating the safety and efficacy of polyclonal FOXP3^+^ Treg cell therapy in various autoimmune disease settings, including type 1 diabetes (NCT02691247) (140–143), lupus (144), multiple sclerosis (145), Crohn’s (146, 147), and Pemphigus vulgaris (NCT03239470), among others. These trials have collectively demonstrated the safety of FOXP3^+^ Treg cell therapy, while additional research is ongoing to further establish their efficacy (**Table 3**).

**Table 3:** Ongoing clinical trials using non-engineered Treg and Tr1 cells in autoimmunity.

Sponsor	Product Name	Product Class	Product Type	Indication(s)	Stage	Clinical Trial Number
**ActiTrexx**	Actileucel	Allogeneic	gp120-stimulated FOXP3 Tregs	GvHD	Phase 1	n.a.
**Cellenkos**	CK0803	Allogeneic	CXCR4 UCB FOXP3 Tregs	ALS​	Phase 2​	NCT05695521
	CK0801	Allogeneic	CXCR4 UCB FOXP3 Tregs	Bone marrow failure​	Phase 1​	NCT03773393
	CK0802	Allogeneic	CXCR4 UCB FOXP3 Tregs	COVID-19 ARDS​	Phase 2​	NCT04468971
	CK0804	Allogeneic	CXCR4 UCB FOXP3 Tregs	Myelofibrosis​	Phase 1​	NCT05423691
**Fundacion Publica Andaluza**	N/A	Autologous	FOXP3 Tregs	GvHD	Phase 1​	NCT05095649
**ORCA Bio​**	Orca-Q​	Autologous	Freshly sorted Treg enriched graft; haplo	GvHD	Phase 1​	NCT03802695
	Orca-T​	Autologous	Freshly sorted Treg enriched graft; full match required	GvHD​	Phase 3	NCT04013685 and NCT05316701
**PolTreg​**	PTG-007​	Autologous	FOXP3​ Tregs	Multiple sclerosis ​	Phase 1​	n.a.
				T1D​	Phase 2​	n.a.
**Stanford University**	T-allo10	Allogeneic	Tr1	GvHD	Phase 1	NCT04640987
**Tract Tx**	TRACT​	Autologous	FOXP3 Tregs​	Kidney transplant​	Phase 2​	NCT02145325

UCB, umbilical cord blood; haplo, haploidentical; ALS, amyotrophic lateral sclerosis; ARDS, acute respiratory distress syndrome; T1D, type-1 diabetes; GvHD, GvHD prevention studies.

Umbilical cord blood has been used as an alternative source of FOXP3^+^ Treg cells for cell therapy. A small-scale trial for GvHD prevention investigated the use of partially matched cord-blood FOXP3^+^ Treg products in five patients undergoing stem cell transplantation (NCT02423915). Although GvHD occurred in all four surviving patients, symptoms resolved, and patients were able to discontinue immunosuppressive medications after a median follow-up of 25 months (148). The safety and potential efficacy of allogeneic cord-blood-derived FOXP3^+^ Treg cells are also being evaluated in an ongoing clinical trial involving six patients with amyotrophic lateral sclerosis (ALS) without HLA matching (NCT05695521). A subsequent Phase 1b trial is currently underway, aiming to assess 60 additional patients in a randomized, controlled study.

A key challenge of natural FOXP3^+^ Treg cell manufacturing has been the potential for these cells to lose FOXP3 expression, particularly when exposed to inflammatory conditions (149), and convert to an undesired effector T cell phenotype (150–153). Conventional CD4^+^ T cells engineered to constitutively express FOXP3 present a compelling alternative to ex-vivo expanded natural FOXP3^+^ Treg cells. Enforced expression of FOXP3 effectively establishes a stable FOXP3^+^ Treg phenotype and associated suppressive functions, even under inflammatory conditions (154–156). The efficacy of these cells, generated using lentiviral-mediated transduction of FOXP3, has been demonstrated in preclinical models of GvHD and IPEX, and is currently being evaluated in a clinical study of patients with IPEX syndrome (NCT05241444) (154, 157). Gene editing technologies, such as CRISPR or TALEN, have also been employed to drive FOXP3 expression and generate engineered FOXP3^+^ Treg cells with a stable phenotype. In the context of IPEX patients, these techniques have been employed to restore functional FOXP3 expression by substituting the mutated and deficient endogenous sequence with a functional version of the gene (155). Alternative gene editing techniques include insertion of a robust enhancer/promoter near the FOXP3 locus (156), or utilization of a dual knock-in/knock-out approach to insert FOXP3 into a specific locus, while simultaneously disrupting expression of the targeted gene (158).

### 4.3 Tr1 Treg cell therapies to treat immune-mediated diseases

In a 2009 study, antigen-specific, clinical-grade Tr1 Treg cell clones were successfully isolated and expanded *in vitro*, and used to treat patients with refractory moderate-to-severe Crohn’s disease (159). This Phase 1/2a trial evaluated the safety and efficacy of autologous, ovalbumin-specific Tr1 Treg cell clones in patients fed with an ovalbumin-rich diet, to ensure cell activation specifically in the gut (160). The therapy was well-tolerated, and early efficacy signals were noted despite documented product impurities and cell exhaustion following an extensive and time-consuming expansion and activation protocol. Other allo-antigen-specific Tr1-enriched cell therapies have been developed and tested in the prevention of GvHD. CD4^+^ T cells co-cultured with CD3-depleted allogeneic PBMCs were manufactured in presence of IL-10, resulting in a product containing 3-5% of Tr1 Treg cells. This cell product was infused in patients undergoing haploidentical HSCT to facilitate immune reconstitution without GvHD (161). The treatment resulted in long term tolerance and cure (follow up > 7 years) in a subset of patients, without inducing general immune suppression, as tolerized patients mounted normal immune responses against microbes and vaccines. More recently, another host alloantigen-specific donor-autologous Tr1-enriched cell product containing 9-13% of Tr1 cells, was used to treat children and adults with hematologic malignancies receiving unmanipulated (NCT03198234) or TCRαβ^+^ T cell and CD19^+^ B cell depleted (NCT04640987) HSCT from mismatched unrelated or haploidentical donors (162). In these two clinical trials, no dose limiting toxicity has been observed in any of the patients treated so far. Patients displayed good T-cell immune reconstitution without increased risk of GvHD. Preliminary analyses suggested a reduced incidence of severe infections compared to historical controls. Furthermore, Tr1 Treg cells were detectable in the peripheral blood shortly after product infusion, reaching the highest levels at day +7 post-infusion and persisting for up to one year after administration (163).

While clinical trials have demonstrated the safety of patient- or donor-derived Tr1 Treg cell therapies, just like with natural FOXP3^+^ Treg cells, generating large numbers of highly pure Tr1 Treg cells from a low-frequency population remains a challenge. To address this limitation, recent efforts have focused on engineering allogeneic CD4^+^ T cells to express IL-10 and inducing cellular reprogramming through specific activation and expansion protocols. The resulting engineered cells effectively recapitulate many aspects of natural Tr1 cell biology, including high IL-10 secretion, low IL-4 production, *in vitro* suppression of T cells, and *in vivo* protection from GvHD in humanized mouse models (164–166). This efficient manufacturing of Tr1-like cells has enabled the development of novel polyclonal, allogeneic, IL-10-engineered Tr1 Treg cell therapies. These therapies are currently in clinical development for the prevention of GvHD following mismatched HSCT (NCT06462365), as well as for the treatment of refractory Crohn’s disease (167).

### 4.4 Opportunities for antigen-targeted, engineered FOXP3^+^ Treg and Tr1 Treg cell and Tr1 cell therapies

To achieve localized immune modulation, improve efficacy, and avoid the risk of systemic, generalized immunosuppression, engineered regulatory T cells can be equipped with a targeting moiety to focus their activity on specific tissues or cell types. The advantage of this approach is supported by evidence that antigen-specific Treg cells exhibit greater potency compared to their polyclonal counterparts (134–137). However, an inherent challenge lies in the typically low frequency of antigen-specific Treg cells, which can complicate the manufacturing process and limit its scalability.

As an alternative, antigen-specific regulatory T cells can be generated by incorporating a CAR or antigen-specific TCR, which facilitates the generation of large numbers of cells. For instance, conventional CD4^+^ T cells have been reprogrammed to co-express FOXP3, an engineered IL-2 receptor responsive to rapamycin, and a pancreatic islet antigen-specific TCR (168). This TCR-FOXP3^+^ Treg cell therapy product is being developed for the treatment of type 1 diabetes, aiming to prevent T cell-mediated destruction of pancreatic islet β cells. Another example comes from HLA-A2-directed CAR FOXP3^+^ Treg cells, which have demonstrated superior potency compared to polyclonal FOXP3^+^ Treg cells, particularly in solid organ transplantation (169–173). Clinical trials are currently underway to evaluate autologous HLA-A2-specific CAR Treg cell therapy in kidney and liver transplantation, aiming to induce immunological tolerance to the transplanted organ and eliminate the need for lifelong immunosuppression (NCT04817774; NCT05234190) (174). An alternative target, CD6, is a cell surface antigen expressed on T lymphocytes and NK cells. An allogeneic, CD6-CAR FOXP3^+^ Treg cell therapy is in development for the treatment of chronic GvHD after HSCT (NCT05993611). Beyond transplantation and GvHD, several other autologous CAR- and TCR-redirected FOXP3^+^ Treg cell therapies are being developed for various autoimmune diseases, including multiple sclerosis (NCT06566261), SLE and lupus nephritis (175, 176), rheumatoid arthritis (NCT06201416), and hidradenitis suppurativa (NCT06361836). Several donor-derived, allogeneic CAR-Treg cell therapies are also in earlier stages of development and are anticipated to enter clinical trials soon. Tr1 Treg cells possess both regulatory and cytotoxic functions (66, 165). Another tantalizing, yet theoretical strategy would be to equip Tr1 Treg cells with a B cell-targeting CAR, enabling them to eliminate autoreactive B cells, while simultaneously suppressing pathogenic T cells and inflammation.

A comprehensive overview of clinical trials involving antigen-targeted, engineered regulatory T cells in autoimmune and immune-mediated diseases is presented in **Table 4**. The relatively small, but growing number of these efforts highlight an increasing interest in harnessing the therapeutic potential of autologous or allogeneic engineered regulatory T cells to treat a wider range of immune disorders.

**Table 4:** Ongoing clinical trials using engineered Treg and Tr1 Cell therapeutics in autoimmunity.

Sponsor	Product Name	Product Class	Product Type	Indication	Phase	Clinical Trial Number
**Abata Tx**	ABA-101​	Autologous	DRB1*15:01-specific TCR FOXP3 Treg​	Multiple sclerosis	Phase 1​	NCT06566261
**City of Hope**	CD6 CAR Treg	Autologous	CD6 CAR FOXP3 Treg	GvHD	Phase 1​	NCT05993611
**Quell Tx**	QEL-001	Autologous	HLA-A2-specific CAR FOXP3 Treg	Liver transplant	Phase 1	NCT05234190
**Sangamo Tx**	TX200-TR101	Autologous	HLA-A2-specific CAR FOXP3 Treg	Kidney transplant	Phase 1	NCT04817774
**Sonoma Biotherapeutics​**	SBT-77-7101​	Autologous	CAR FOXP3 Treg​	Rheumatoid Arthritis​	Phase 1​	NCT06201416
				Hidradenitis suppurativa​	Phase 1​	NCT06361836
**Stanford University**	CD4^LVFOXP3^	Autologous	FOXP3 Treg	IPEX	Phase 1	NCT05241444
**Tr1X**	TRX103	Allogeneic	IL10-engineered Tr1	GvHD	Phase 1	NCT06462365
				Crohn’s	IND cleared	n.a.

GvHD, GvHD prevention study; IPEX, Immune Dysregulation Polyendocrinopathy Enteropathy X-linked syndrome.

## 5 Limitations and opportunities

### 5.1 Moving beyond lymphodepleting conditioning regimens

Lymphodepletion, commonly achieved through the combined administration of fludarabine and cyclophosphamide, is a standard conditioning regimen employed in CAR T cell therapies for both cancer and autoimmune diseases. This procedure is crucial for enhancing the engraftment, expansion, and persistence of the infused CAR T cells. However, it is associated with significant toxicity, including prolonged cytopenia and increased susceptibility to infections (177–183). While lymphodepletion is generally well-tolerated in the context of oncology, its application in autoimmune diseases raises concerns due to the potential for exacerbating the underlying condition through severe side effects. Interestingly, a case study involving a patient with SLE suggests that CAR T cell therapy may be effective in autoimmune diseases even with reduced doses of lymphodepleting agents (184). This observation may indicate that the threshold for achieving therapeutic efficacy in autoimmune diseases might be lower than in oncology, opening the door for lower, less aggressive lymphodepletion regimens. Reducing the intensity of conditioning regimens may necessitate strategies to enhance T cell expansion and compensate for lower peak CAR T cell numbers, a factor strongly correlated with efficacy in oncology (181, 183). Various engineering strategies aimed at augmenting T cell expansion and function will be explored below.

For Treg-based cell therapies, the IL-2 signaling pathway plays a critical role in the homeostasis and function of FOXP3^+^ Treg cells, therefore, augmenting IL-2 signaling represents a promising strategy for enhancing this type of cell therapy. Early clinical investigations employing IL-2 as an adjuvant for CAR T cell therapy in non-Hodgkin lymphoma demonstrated an increase in the frequency of FOXP3^+^ Treg cells (185, 186). This observation is consistent with the well-established role of IL-2 in promoting the survival, stability, and suppressive function of FOXP3^+^ Tregs (187, 188). Furthermore, clinical trials exploring low-dose IL-2 administration in patients with autoimmune diseases have consistently reported an expansion of FOXP3^+^ Treg populations, although the overall clinical responses have been variable (189). However, a significant limitation of systemic IL-2 administration, even at low doses, is its potential to activate other immune cell subsets and induce severe side effects, thus restricting its utility as an adjuvant for cell therapy (190). To circumvent this challenge, innovative orthogonal IL-2 systems have been developed. These systems utilize a modified IL-2 molecule that selectively binds to an engineered IL-2 receptor, enabling the targeted expansion of cells expressing this artificial receptor (191, 192). Preclinical studies have demonstrated the efficacy of this approach. FOXP3^+^ Tregs engineered to express the orthogonal IL-2 receptor can be selectively expanded *in vivo* using orthogonal, soluble IL-2, leading to improved donor hematopoietic stem cell engraftment and organ transplant tolerance (193). While this orthogonal IL-2 technology is currently undergoing clinical development in the field of oncology (NCT05665062), its therapeutic potential for autoimmune diseases warrants further investigation.

Beyond IL-2, other cytokine pathways offer promising targets for enhancing the *in vivo* proliferation and persistence of therapeutic effector and regulatory T cells. To mitigate the risk of exacerbating autoimmune pathology through systemic cytokine exposure, a favored strategy is to engineer T cells to co-opt the pathway in a cell-intrinsic fashion (194). Homeostatic cytokines such as IL-7 and IL-15 are well-known for their ability to augment the persistence and function of effector T cells. Notably, the efficacy of lymphodepletion is partially attributed to the increased availability of these cytokines (195). Preclinical studies have demonstrated that engineering CAR T cells to express a constitutively active form of the IL-7 receptor enhances their proliferation and persistence *in vivo* (196, 197). Similarly, co-expressing CAR with membrane-bound IL-15 has been shown to improve CAR T cell expansion and persistence in murine models (198), with encouraging clinical results reported in an oncology setting (199). Given the critical role of IL-15 in the maintenance and expansion of Tr1 Treg cells (200), IL-15 overexpression could also prove beneficial for Tr1-based therapies. While IL-12 and IL-18 have demonstrated potential in promoting CAR T cell proliferation and persistence without preconditioning in murine models (201, 202), their pro-inflammatory nature poses a significant challenge for their application in autoimmune diseases.

The pleiotropic nature of IL-10 presents a unique advantage for CAR T cell therapy in autoimmune diseases. IL-10 can enhance the persistence of engineered T cells through autocrine signaling while simultaneously promoting an anti-inflammatory response via paracrine signaling. Studies have shown that IL-10 improves CAR T cell proliferation and mitochondrial health, a critical determinant of T cell fitness (203). Moreover, IL-10 contributes to memory CD4^+^ T cell homeostasis and has been linked to the prolonged persistence of CD4^+^ T cells in the central nervous system during viral infections (204, 205). Importantly, Tr1 Treg cells, which naturally produce IL-10, can transfer a Tr1-like phenotype to bystander CD4^+^ T cells, a positive feedback loop known as infectious tolerance (206, 207). Through these combined mechanisms, it is plausible that Tr1-based therapies may require less expansion *in vivo* compared to effector CAR T cells to achieve comparable clinical benefit.

### 5.2 Long-term toxicity/safety of B-cell targeting effector CAR T and CAR Treg cells

Beyond the immediate risks associated with lymphodepletion, careful consideration must be given to the long-term safety of B-cell targeting CAR T cell therapies, particularly the potential for prolonged B cell aplasia and its impact on humoral immunity. In the context of cancer treatment, extended B cell depletion can increase the risk of infections and compromise B cell memory responses, with studies reporting a significant incidence of pneumonia requiring hospitalization months after CAR T cell infusion (208, 209). However, current treatment protocols for autoimmune diseases appear to result in the rapid clearance of CAR T cells, potentially mitigating this risk (61, 62). This observation is supported by clinical data demonstrating a low incidence of serious infections following CAR T cell therapy, despite profound and prolonged B cell depletion, lasting more than 100 days in some patients (62). It is crucial to recognize that these risks may be more pronounced with BCMA-targeting CARs compared to CD19-targeting CARs, due to the distinct B cell subpopulations expressing these antigens (81–83). Additionally, the risk of excessive immunosuppression warrants particular attention for non-targeted regulatory T cell therapies, as FOXP3^+^ Treg cells are known to suppress immune responses to various pathogens and malignancies (210, 211). Therefore, as the field progresses, it will be essential to develop strategies for controlling the proliferation of therapeutic FOXP3^+^ T cells, particularly those of autologous origin. Incorporating “kill switch” or suicide gene technology may enable the controlled elimination of these cells once the therapeutic objective is achieved.

One widely employed approach is the inducible caspase 9 (iCas9) system, which has been utilized in numerous clinical trials (212). Activation of iCas9 triggers apoptosis, leading to the elimination of the engineered T cells. Another strategy involves the herpes simplex virus thymidine kinase (HSV-tk) suicide gene, which converts the prodrug ganciclovir into a toxic metabolite, disrupting DNA synthesis and inducing cell death (213). However, the potential immunogenicity of HSV-tk can lead to premature elimination of the therapeutic cells by the host immune cells (214). Alternatively, co-expressing a truncated, inactive form of EGFR (EGFRt) or CD20 on the surface of engineered cells allows for their targeted elimination through antibody-dependent cellular cytotoxicity or complement activation upon administration of clinically approved monoclonal antibodies such as cetuximab or rituximab (215, 216).

While these safety mechanisms have primarily been explored in CAR T cell therapies for cancer, there is growing interest in incorporating them into engineered Treg cell therapies as well (217). This is exemplified by an ongoing Treg-based clinical trial employing an undisclosed safety switch technology (NCT05234190).

While suicide switch technologies offer effective elimination of engineered T cells, complete eradication may be disadvantageous for CAR Treg cell therapies, where maintaining a reservoir of potentially beneficial cells could be desirable. Positive regulators of CAR activity provide an alternative approach, enabling future reactivation of the therapeutic cells if needed. These systems utilize small molecules or drugs to control the expression or activity of the CAR transgene. This control can be implemented at the genetic level, using inducible promoters to regulate CAR expression, or at the protein level, through various mechanisms (comprehensively reviewed in (218, 219)). An example of such an inducible system is currently being evaluated in a clinical trial for acute myeloid leukemia (NCT05105152), where rapamycin acts as a dimerization agent to activate CAR functionality (220, 221). These inducible systems provide an “on-off” switch for CAR T cell activity, allowing for treatment to be tailored to the patient’s disease activity and offering greater control and flexibility. It is important to note that donor-derived allogeneic T cell therapies are naturally subject to rejection by the host immune system over time. Therefore, this class of therapeutics is expected to pose a lower risk of long-term adverse effects.

### 5.3 Scaling the production of cell therapies for autoimmunity

The scalability and cost of manufacturing autologous T cell therapies, including Treg, Tr1, and CAR T cell therapies, has been a major obstacle to their widespread application beyond rare diseases like acute lymphoblastic leukemia or multiple myeloma. The prospect of treating more prevalent autoimmune diseases, such as lupus and multiple sclerosis, which affect millions of patients worldwide, poses significant manufacturing, logistical, and financial hurdles. Furthermore, patients with autoimmune diseases often require long-term treatment with glucocorticoids or other immunosuppressive agents, particularly when their disease is poorly controlled. These medications can negatively impact T cell number and function, potentially compromising the collection, manufacture, and quality of the final autologous T cell product. Allogeneic, or “off-the-shelf”, cell therapies, where cells from universal donors are engineered for multiple patients, offer a potential solution to the inherent limitations of autologous cell therapy. However, allogeneic T cell therapy faces significant challenges, including the risk of GvHD due to the alloreactive TCR repertoire of the infused cells, and the potential for rapid rejection mediated by host CD8+ T cells and NK cells (222).

The advent of gene editing technologies, particularly CRISPR-Cas9 and its derivatives, has ushered in a new era for allogeneic T cell therapies. These tools enable precise modifications of T cells, mitigating the risk of GvHD by directly eliminating TCR expression and enhancing their persistence in non-HLA identical recipients by ablating HLA expression, thereby reducing the likelihood of rejection by the host immune system. The clinical potential of multiplexed gene editing with CRISPR-Cas9 has been demonstrated in several effector CAR T cell clinical trials in oncology (NCT04502446) (223–226). Consequently, numerous allogeneic CD19-targeting CAR T cell therapies are being actively developed for the treatment of autoimmune diseases (**Table 1**). One such product utilizes CRISPR-Cas9 to simultaneously knock-out the TCR α chain to prevent GvHD, as well as HLA-A/B, CIITA, and PD-1 to evade host-mediated immune rejection and enhance persistence (66). This therapy is currently in clinical development for myositis and systemic sclerosis, where it has shown promising results, achieving sustained remission in three out of three patients (NCT05859997). Other clinical studies with gene-edited CAR T cells are also underway in lupus and ANCA-associated vasculitis (NCT06308978, NCT06294236). It is worth noting that multiplexed gene editing using CRISPR-Cas9 may come with risks due to unpredictable translocation events between double-stranded breaks (227). Future strategies using Cas9-derived “base editors” hold promise to greatly improve the safety profile of edited allogenic products. These variants may enable the targeted editing of specific genetic loci without inducing genotoxic breaks in the DNA (228). The improved safety profile and efficacy of this approach has been demonstrated preclinically and in a Phase 1 study with quadruply-edited allogeneic CAR-T cells for relapsed leukemia showing undetectable translocation events (229, 230). As an alternative to using genetic editing to knock-out TCR expression, generating allogeneic CAR T cells with a virus-specific, non-alloreactive TCR may reduce the risk of GvHD and simplify the manufacturing process, an approach being tested clinically for patients with SLE (NCT06429800).

Unlike allogeneic effector CAR T cells, which require extensive genetic engineering to prevent GvHD and rejection, Tr1 and FOXP3^+^ Treg cells possess inherent suppressive properties that naturally mitigate their immunogenicity and potential to induce toxicity via GvHD. To date, there is no evidence to suggest that the transfer of allogeneic Tr1 or FOXP3^+^ Treg cells induces GvHD in humans. For instance, a recent Phase 1 study of HLA-mismatched, cord-blood derived Treg cells used to treat nine patients with bone marrow failure syndrome showed no signs of GvHD or other serious side effects (231). Moreover, IL-10 production by Tr1 cells can directly and indirectly suppress alloantigen-specific T cell responses (232), potentially enhancing their persistence *in vivo* (NCT06462365). Development of an allogeneic cell therapy that does not necessitate extensive gene editing would greatly simplify manufacturing complexities and dramatically lower its costs. This opens the door for broader development of allogeneic Treg-based cell therapies as a way to obviate manufacturing and scalability challenges.

## 6 Discussion

Over the last thirty years, the treatment of autoimmune and inflammatory diseases has undergone a remarkable transformation, progressing from broadly immunosuppressive drugs to targeted biologics and now to the promising field of cell therapies. HSCT, while effective in severe cases, carries significant risks and has limited applicability. MSCs offer a less invasive option but face challenges in standardization, persistence and in achieving consistent clinical outcomes.

The success of CAR T cell therapy in oncology has paved the way for its exploration in autoimmunity, with promising early clinical trials showcasing the safety and efficacy of B-cell-targeted CAR T cells in various conditions. The prospect of inducing long-term remission through an immune system reset offers hope for a long-lasting, if not curative, outcome. However, further validation is needed through larger-scale clinical trials with extended follow-up periods. These studies will be crucial for confirming the long-term safety and efficacy of this approach in a broader patient population and for assessing the durability of treatment responses. Treg and CAR Treg cell therapies, which suppress pathogenic T cells and inflammation, offer a potentially safer and more effective approach for treating diseases characterized by dysregulated T cell activation and inflammation, such as psoriasis or rheumatoid arthritis. Furthermore, combining the immunosuppressive properties of Treg cell therapies with a B-cell suppressing modality may offer a promising new avenue for treating diseases caused by both pathogenic B and T lymphocytes, such as multiple sclerosis.

The path forward for these promising therapies involves addressing challenges such as minimizing the need for lymphodepletion, ensuring long-term safety, and enhancing scalability, lower cost and increase accessibility. Onerous autologous approaches continue to pose a significant hurdle for their broader application in autoimmune diseases with higher prevalence than subtypes of cancer. Allogeneic therapies, coupled with gene engineering or gene editing technologies like CRISPR-Cas9, offer a potential solution by overcoming the limitations of personalized treatment and the risk of GvHD or rejection, although achieving sufficient cell persistence remains a concern.

The future of cell therapies in autoimmune and immune-mediated diseases holds immense promise in the quest to alleviate patients’ lifelong reliance on immunosuppressants. Ongoing research and clinical trials are laying the groundwork for the development of next-generation therapies that are safer, more effective, and scalable. The potential to achieve long-term remission or even cures, coupled with a deeper understanding of the complex mechanisms governing immunological tolerance offers hope for a transformative shift in the treatment of these diseases.
